# The Passive Strategy During a Sustained Attention to Responses Task (SART): How Does the Mind Divide Its Mental Resources During a Simple, Repetitive Task?

**DOI:** 10.1192/j.eurpsy.2026.11612

**Published:** 2026-07-31

**Authors:** S. Ray, H. Witchel

**Affiliations:** Brighton and Sussex Medical School, Brighton, United Kingdom

## Abstract

**Introduction:**

Mind wandering (MW) is a mental state in which attention drifts away from the task at hand. While MW is known to increase errors in monotonous tasks, less is understood about the attentional strategies used during sustained, repetitive tasks.

**Objectives:**

We investigated whether participants adopt a distinct “passive strategy” that combines both slower reaction times and increased error rates.

**Methods:**

A total of 103 healthy participants were recruited online via Prolific. Participants completed a Sustained Attention to Response Task (SART) built on Gorilla.sc, in which digits (1–9) were presented sequentially with varying proportions of no-go trials (4%, 25%, 75%, 100%). Participants responded to digits other than “3” and withheld responses to “3.” After each block, they completed thought probes and subjective ratings (difficulty, effort, caution, double-checking, passivity). Data was analysed using t-tests and linear mixed-effects models (LMEs).

**Results:**

The 4% press percentage condition (PP04) produced the slowest mean reaction times, the highest omission error rates, and the lowest commission error rates. Subjective ratings indicated PP04 was also the most passive condition. This pattern differs from previously proposed attentional strategies, which predict either higher error rates with faster responding or slower reaction times with greater accuracy.

**Conclusions:**

Persistent inhibition during sparse no-go conditions induced a “passive strategy,” characterized by both increased omission errors and slower reaction times. This strategy may have clinical and occupational implications for error-prone repetitive tasks, such as patient monitoring, medication delivery, and documentation - key tasks for psychiatrists. Future experiments can be carried out with individuals with attention deficit hyperactivity disorder (ADHD) to see if there is any increased passivity or error-making under the same conditions.

**Disclosure of Interest:**

None Declared

